# A Novel Approach for More Effective Emergency Equipment Storage: The Task-Based Package-Organized Neonatal Emergency Backpack

**DOI:** 10.3389/fped.2021.771396

**Published:** 2021-12-23

**Authors:** Lorenz Sommer, Mercedes Huber-Dangl, Katrin Klebermaß-Schrehof, Angelika Berger, Eva Schwindt

**Affiliations:** Department of Pediatrics and Adolescent Medicine, Division of Neonatology, Pediatric Intensive Care and Neuropediatrics, Comprehensive Center for Pediatrics, Medical University Vienna, Vienna, Austria

**Keywords:** neonatology and pediatric intensive care, emergency equipment and supply, human factors, emergency and critical care, crew resource management (CRM)

## Abstract

**Objectives:** To evaluate a new task-based package-organized (TPO) neonatal emergency backpack and to compare it to the classical (ABC- and material-based) backpack.

**Methods:** Simulation-based assessment of time to retrieve equipment for three different tasks [intraosseous access (IO), intubation and adrenaline administration] using the TPO and the classical emergency backpack was compared.

**Results:** Equipment retrieval times for the three tasks were assessed for 24 nurses (12 intermediate care, 12 intensive care) and were significantly faster in the TPO than in the classical backpack (IO 33 vs. 75 s, *p* < 0.001; intubation 53 vs. 70 s, *p* = 0,001; adrenaline 22 vs. 45 s, *p* < 0.001). The number of missing items was significantly lower using the TPO backpack for IO and adrenaline retrieval (IO 0,9 vs. 2,3 items, *p* < 00001, adrenaline 0.04 vs. 1, *p* < 0.001) but not for intubation equipment (0.9 vs. 1, not significant). The subjective rating of overall clearness was significantly higher for the TPO compared with the classical backpack (5,9 vs. 3,5, *p* < 0.001).

**Conclusion:** Task-based package organization of neonatal emergency backpacks is feasible and might be superior to ABC-/material-oriented storage.

## Introduction

Given that “proper preparation prevents poor performance,” a well-planned emergency kit with all potentially required material is of paramount importance in critical medical events. This is even more true for emergencies outside the “home ward” or even preclinically. Emergency kits, such as trolleys or backpacks, are used for in-hospital neonatal emergencies. However, thus far, published literature on recommendations for the content of neonatal emergency kits is scarce, and authors rarely focus on *how* to arrange emergency equipment (1). López-Herce Cid et al. (2) provide a list of recommended equipment in a neonatal emergency kit. Regarding “how” to store these items, they recommend “it should be organized so that equipment and medication can be found easily and intuitively.” Unfortunately, these authors do not provide any suggestions on how to do so, and to our knowledge, there is no other study concerning this topic.

Whether found on an emergency cart or trolley or in a backpack, emergency equipment is often stored according to categories of material, such as “syringes” or “tubes.” Other approaches include ABC (airway—breathing—circulation)-oriented storage, or material is stored in an escalating manner in accordance with the recent Neonatal Resuscitation Program (NRP) guidelines from the American Academy of Pediatrics (3). A common problem with all these approaches is the requirement to retrieve the complete equipment for one task from several drawers or bags because consumables, such as syringes or luer-lock caps, are often stored in other locations. This retrieval process not only wastes valuable time during an emergency event but also supposedly leads to higher stress levels in medical teams in an already very stressful situation and hence might impede resuscitation performance.

Standardized procedures and required equipment however could already be considered when designing space for equipment storage. Therefore, we developed a task-based package-oriented (TPO) emergency backpack with the goal of storing and packing together equipment for each specific task without the need to retrieve any additional material from other bags. In neonatal units, this approach is often implemented in prepacked sets for umbilical venous catheters (4).

In this study, we aimed to evaluate the hypothesis that equipment retrieval is faster and easier for medical teams with TPO emergency backpacks than for those with conventional material-oriented backpacks.

## Methods

For this cross-over study bedside nursing staff from two IMCs and two ICU wards (both Children's University Hospital, Medical University Vienna) were randomly recruited. Power calculation (G^*^Power 3.1 for Mac, Apple Inc., USA) was based on an estimated difference of 15 s for retrieval times and revealed 24 participants. Therefore, six nurses were recruited from each ward. The nurses were asked to retrieve equipment for (1) intraosseous access (IO), (2) intubation and (3) epinephrine administration once using the classical emergency kit and once using the TPO emergency kit. The assessment began at 50% with the classical kit and at 50% with the TPO kit. The first three nurses were asked to begin with the classical kit and the last three nurses with the TPO kit—vice versa with the other IMC/ICU ward. An initial explanation was not provided for either emergency kit (classical or the new TPO kit). All assessments were performed on the ward. Primary outcome was the time required for equipment retrieval, which was measured using a standard stopwatch. Secondary outcome was the potential impact of influencing factors, which were assessed via questionnaire. This questionnaire was completed by all participants to obtain the following information:

- Previous experience in neonatal care (years)
- Working experience at the Children's University Hospital of the Medical University Vienna
- Previous experience with the classical emergency kit in real-life emergency events
- Self-evaluation of estimation of confidence in handling using the classical emergency kit (scale 1 to 6, with 1 = not very confident and 6 = very confident)
- Individual rating of overall arrangement of both emergency kits (scale 1 to 6, with 1 = very unclear and 6 = very clear arrangement)
- Assessment of frequency on how often individuals familiarize themselves with the emergency kit (weekly, monthly, 1–2 times every 6 months, 1–2 times every 12 months or never).

### Classical vs. TPO (Task-Based Package-Organized) Emergency Kit

**Figure 1:**
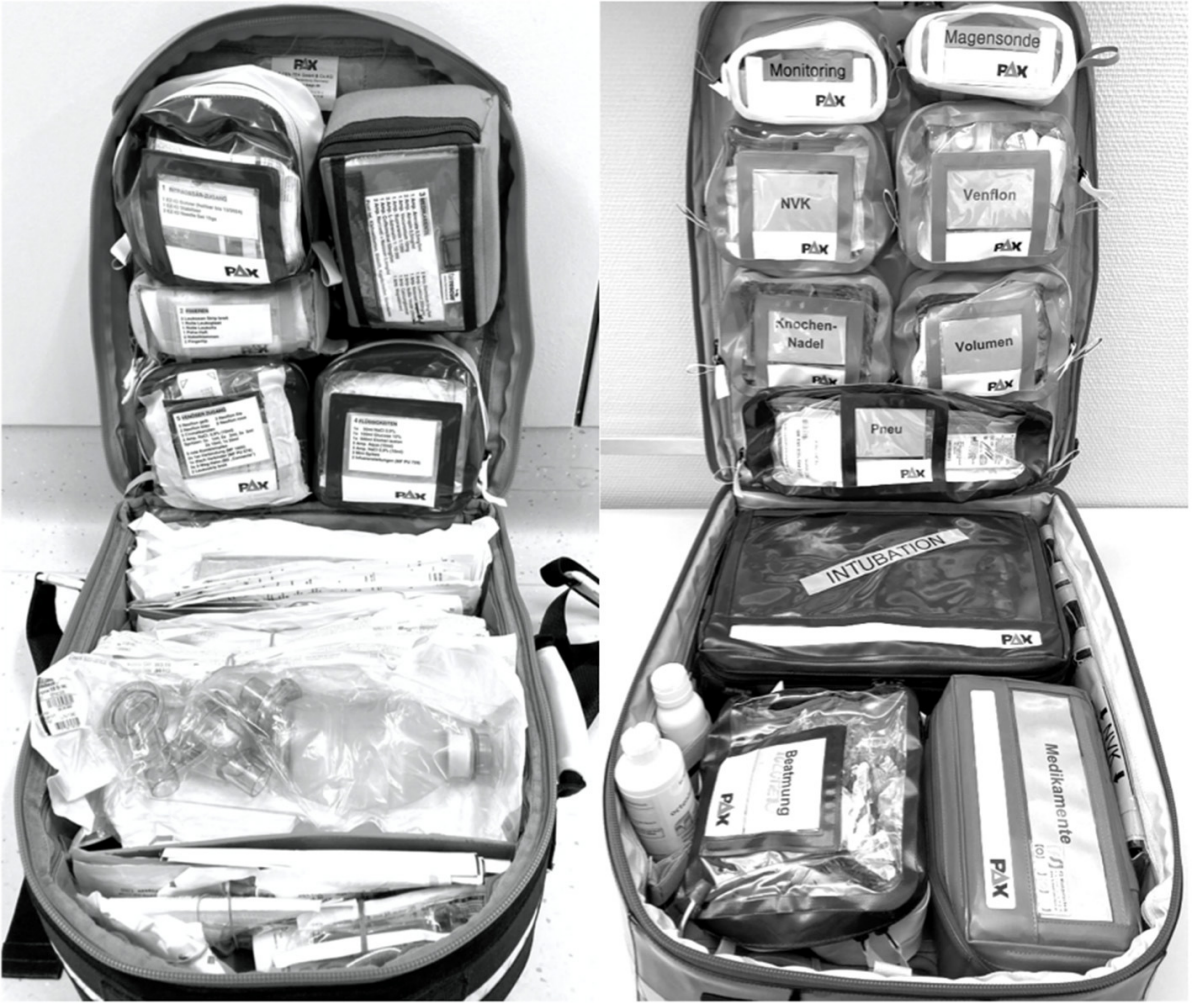
Classical emergency backpack **(left)** and task-based package-organized backpack **(right)**: Magesonde = gastric tube, NVK = UVC (umbilical venous access), Venflon = peripheral venous access, Knochennadel = intraosseous access, Volumen = fluids, Pneu = pneumothorax, Beatmung = ventilation, Medikamente = medication.

The classical emergency kit is an emergency backpack from X-CEN-TEK GmbH & Co KG (Wardenburg, Germany); see Figure 1; Table 1. Equipment is stored partly based on an ABC-oriented approach (i.e., “airway”), partly according to type of material (i.e., “plasts”) and partly using a task-based approach (i.e., “venous access”). Due to this mixture of packaging types, even in task-based oriented bags, the necessity to retrieve for multiple bags arises to compile all required equipment for one task. For example, the storage unit labeled “medications” contains all necessary medications, but syringes must be retrieved from a storage unit labeled “venous access.”

**Table 1:** Item list of the task-based package-organized emergency backpack.

	**Quantity**		**Quantity**
**Monitoring**		NaCl 0,9% syringe 5 or 10 ml	2
ECG-electrodes term newborn	1	Leukoplast 1 cm	1
ECG-electrodes preterm	1	SET^*^ IO:	
Sensor for saturation term newborn	1	EZ-IO needle red (15 mm, 15 GA)	1
Sensor for saturation preterm	1	Swab sterile 5 x 5 cm	1
Posey	2	Three-way stopcock luer lock	1
**Gastric tube**		NaCl 0,9% syringe 5 or 10 ml	1
Gastric tube Fr 4	2	**Umbilical venous access**	
Gastric tube Fr 6	2	UVC 2,5 Fr single lumen	1
Enteral syringe 10 ml	2	UVC 5 Fr double lumen	1
Leukoplast 1 cm	1	Sterile drape 50 x 50 cm	1
**Peripheral venous access**		Acutenaculum 16 cm	1
Neoflon 26 GA	4	Tweezers anatomic 14 cm	1
Neoflon 24 GA	4	Tweezers surgical	1
Venflon 22 GA	2	Scalpel Nr. 11	1
Swab sterile 5 x 5 cm	4	Stitch cutter	1
Syringe 2 ml	4	Sutures 2–0 silk 45 cm	1
NaCl 0.9% 10 ml	2	Swab sterile 10 x 10 cm	2
Three-way stopcock luer lock	2	Swab sterile 5 x 5 cm	5
Extension line single	2	Steristrips white	2
Steristrips white	2	Leukoplast 2.5 cm	1
Blood gas analysis	2	Syringe 2 ml^**^	2
Cap Luerlock red	5	NaCl 0,9% 10 ml^**^	1
Blunt cannula 18 GA	5	Blunt cannula 18 G^**^	1
PehaHaft 4 cm	1	Cap Luerlock red^**^	2
Sterile scissors	1	**Ventilation**	
SET^*^ PVC:		Ventilation bag 500 ml	1
Neoflon 26 GA	2	Reservoir	1
Neoflon 24 GA	2	Oxygen line	1
Swab sterile 5 x 5 cm	2	Mask size 0	1
Syringe 2 ml	2	Mask size 1	1
NaCl 0,9% 10 ml	1	Mask size 2	1
Three-way stopcock luer lock	1	**Medication**	
Extension line single		Flumazenil 0.5 mg/5 ml	1
Steristrips white	1	Aqua ad injectionem 10 ml	3
Blood gas analysis	1	Atropine 0.5 mg/1 ml	2
**Desinfection/auscultation**		Calciumgluconat	1
Octenisept 250 ml	1	Esketamine 25 mg/5 ml	1
Isozid 100 ml	1	L-Adrenalin 2 mg/20 ml	2
Stethoscope	1	Phenobarbital 200 mg/1 ml	1
**Fluids**		Magnesiocard 10 ml	1
Elomel Isotone 500 ml	1	Midazolam 5 mg/5 ml	2
Glucose 10% 100 ml	1	NaCl 0.9% 10 ml	3
Syringe 10 ml	3	Naloxone 0.4 mg/1 ml	1
Syringe 20 ml	3	Natriumbicarbonat 1 molar 20 ml	1
Mini-Spike	2	Phenylephrine Aguettant syringe 50 μg/ml	1
Infusion line with filter	1	Vecuronium bromide 10 mg	1
EZ-IO semiautomatic power drill	1	Aqua a.i. 10 ml	1
EZ-IO Nadel red (15 mm, 15 GA)	2	Suprarenin (adrenaline) 1 mg/1 ml	1
Tupfer steril 5 x 5 cm	4	Syringe 1 ml	6
Three-way stopcock luer lock	2	Syringe 2 ml	5
Syringe 5 ml	3	Cathejell	1
Syringe 10 ml	3	Tube uncuffed 2.0	2
Blunt cannula 18 GA	11	Tube uncuffed 2.5	2
Cap Luerlock red	5	Tube uncuffed 3.0	2
Three-way stopcock luer lock	5	Tube uncuffed 3.5	2
Mini-Spike	1	Tube uncuffed 4.0	2
Marker black	1	Tube uncuffed 4.5	2
Adhesive labels blank (sheet)	1	Guedel 30 mm	1
Adhesive labels “L-Adrenaline”	2	Guedel 40 mm	1
SET^*^ Adrenaline		Guedel 50 mm	1
L-Adrenaline 2 mg/20 ml	2	Guedel 60 mm	1
Syringe 10 ml	1	Guedel 70 mm	1
Syringe 1 ml	1	Suction catheter CH 6	2
Three-way stopcock luer lock	1	Suction catheter CH 8	2
Blunt cannula 18 GA	1	Suction catheter CH 10	2
Adhesive labels “L-Adrenaline”	2	Suction catheter CH 16	2
**Gloves**		Fingertip	1
Gloves sterile 6.0	1	Tendernose	1
Goves sterile 6.5	1	Leukoplast 1 cm	1
Gloves sterile 7.0	1	Steristrips white	1
Gloves sterile 7.5	1	Scissors	1
Gloves sterile 8.0	1	**Pneumothorax**	
Gloves sterile free of latex 6.0	1	Cook Pigtail Drain 5 Fr	1
Gloves sterile free of latex 6.5	1	Cook Pigtail Drain 8.5 Fr	1
Gloves sterile free of latex 7.0	1	Cook Pigtail Drain 10.2 Fr	1
Gloves sterile free of latex 7.5	1	Venflon 20 GA	2
Gloves sterile free of latex 8.0	1	Sterile drape 50 x 50 cm	1
**Airway/Intubation**		Acutenaculum 16 cm	1
Blade straight size 0	1	Tweezers anatomic 14 cm	1
Blade straight size 1	1	Scalpel Nr. 11	1
Blade curved size 0	1	Stitch cutter	1
Blade curved size 1	1	Suture 2–0 silk 45 cm	1
Magill's forceps	1	Swab sterile 10 x 10 cm	2
Laryngoscope	1	Swab sterile 5 x 5 cm	5
Extra batteries Type AA	2	Cosmopor E	4
Laryngeal mask AMBU Aura Gain 1	1	Leukoplast 1 cm	1
iGel size 1	1	Syringe 5 ml	2
Mandrin	1	Syringe 10 ml	2
Pedicap 1–15 kg	1	NaCl 0.9% 10 ml	2
NaCl 0.9% 10 ml	1	Blunt cannula 18 GA	2

*Item list for TPO emergency backpack*.

* *SETs are packed together with a rubber band*.

** *Packed together in nursing bottle*.

In contrast to the classical emergency kit, the TPO backpack is thoroughly ordered according to certain tasks [monitoring, gastric tube, peripheral venous access, IO access, umbilical venous catheter, ventilation, airway/intubation, medication (including epinephrine set), pneumothorax; see Figure 1; Table 2]. There are bags for each task, each containing the complete equipment required for one certain task—and nothing else. Furthermore, some task-based oriented bag subsets are used. For example, the bag labeled “peripheral venous access” contains all requirements for venous access, including extra material and different needle sizes, and, in addition, equipment required for ONE attempt of peripheral venous access is packed together (using a rubber band) within this bag. Also, material is not stored in loose pockets to avoid a disordered arrangement.

**Table 2:** Item list of the classical emergency backpack.

	**Quantity**		**Quantity**		**Quantity**
**Bag No.1 Intraosseous access**		Neoflon 24 GA	5	Extra batteries	2
EZIO-semiautomatic power drill	1	Neoflon 26 GA	3	Blade straight size 0	1
EZIO stabilizer	1	Venflon 22 GA	2	Blade straight size 1	1
Pediatric needle red 15GA	2	Venflon 20 GA	2	Magill's forceps small	1
**Bag No. 2 Plasts**		NaCl 0,9% 10 ml Ampulla	2	Magill's forceps large	1
Leukosan strip	3	Blunt Cannula 18 GA	5	Mandrin	1
Leukoplast	1	Syringe 1 ml	5	Tube size 2.0	2
Leukofix	1	Syringe 2 ml	5	Tube size 2.5	2
Peha Haft	1	Syringe 5 ml	3	Tube size 3.0	2
Umbilical cord clamp	2	Syringe 10 ml	2	Tube size 3.5	2
Fingertip	2	Syringe 20 ml	1	Tube size 4.0	2
**Bag No. 3 Medication**		Cap Luerlock red	5	Tube size 4.5	2
Flumazenil 0.5 mg/5 ml	1	Single connector line	2	Guedel-tube size 00	1
Atropine 0.5 mg/1 ml	2	Fourfold connector line	1	Guedel-tube size 0	1
Vecuronium bromide 10 mg	1	Three-way stopcock luer lock	3	Guedel-tube size 1	1
Suprarenin (adrenaline) 1:1000	1	Leukosan strip	2	Suction catheter Ch 6	1
L-Adrenalin 1:10 000	3	**Pocket No. 1 Catheter/drains**		Suction catheter Ch 8	1
Caffeine citrate 50 mg/5 ml	1	Syringe 50 ml	2	Suction catheter Ch 10	1
Naloxone 0.4 mg/ml	2	Pigtail Cook Drain 5 Fr	1	Suction catheter Ch 16	1
Midazolam 5 mg/5 ml	2	Pigtail Cook Drain 8,5 Fr	1	Tendernose	1
Phenobarbital 200 mg/ml	1	Pigtail Cook Drain 10,2 Fr	1	**Pocket No. 3**	
Esketamine 25 mg/ml	1	Umbilical venous catheter 2,5 Fr	1	Ventilation mask size 0	1
Natriumbicarbonat 1 molar 20 ml	2	Umbilical venous catheter 5 Fr	1	Ventilation mask size 1	1
Calciumgluconate	1	Sterile drape 50 x 50 cm	1	Ventilation mask size 2	1
Magnesiocard	1	Sterile gloves sizes 6,5	1	Ventilation bag 500 ml	1
Post-its	1	Sterile gloves sizes 7	1	Reservoir	1
Pencil	1	Sterile gloves sizes 7.5	1	Oxygen line	1
Pen	1	Sterile gloves sizes 8	1	Stethoscope	1
Adhesive labels (blank)	6	Sterile gloves single use (L)	6	**Pocket No. 4**	
**Bag No. 4 Fluids**		Acutenaculum	1	ECG electrodes	2
NaCl 0.9% 50 ml	1	Sterile scissors	1	Saturation sensor	2
Glucose 10% 100 ml	1	Tweezers anatomical	1	Posey	1
Elomel isotone 500 ml	1	Tweezers surgical	1	Gastric tube Ch 4	2
NaCl 0.9% 10 ml ampulla	3	Scalpel Nr. 11	1	Gastric tube Ch 6	2
Aqua 10 ml ampulla	3	Stitch cutter	2	Syringes enteral 10 ml	2
Mini-spike	3	Sutures silk 2/0	2	Octensisept	1
Infusion line	2	**Pocket No. 2 Airway**		Sterile swabs 5 x 5 cm	5
**Bag No. 5 Venous access**		Laryngoscope	1	Sterile swabs 10 x 10 cm	2

*In this table Item list of the classical emergency backpack. Bags are zipper-closed. Pockets are open with loose arrangement of equipment*.

### Statistics

Descriptive statistics were performed using SPSS Statistics Version 23.0 (IBM). For comparison of items, such as duration or clearness of arrangement, a dependent sample *t*-test was used. Statistical analysis of retrieval times was performed by Kruskal-Wallis rank-sum test to correct for non-normal distribution. Correlation calculations were performed using the Pearson correlation. Level of significance was defined at *p* = 0.05.

This study was conducted under the approval of the ethics committee and the data protection committee of the Medical University Vienna.

## Results

Twenty-four nurses were recruited to participate in the study: 12 from intermediate care (IMC) and 12 from the intensive care unit (ICU). The ICU nurses had a mean of 13.5 (0.5-32) years of experience in neonatal medicine, and the IMC nurses had 9.5 years of experience (0.5–28; *p* = 0.375). The average number of years worked in our hospital was 13.5 years (range 0.5–32) for the ICU staff and 6.6 years (range 0.5–20; *p* = 0.08) for the IMC staff. In total, 96% of the participants were female (23/24). Seventy-five percent of both ICU (9/12) and IMC (9/12) nurses declared to have previously worked with the emergency kit in real-life emergency events.

Regarding the frequency of how often nurses familiarized themselves with the emergency kit, two nurses (8.3%) declared “never,” 13 (54.2%) “1–2 times per year,” eight (33.3%) “once a month” and zero reported “once a week.” The self-evaluation of confidence in overall handling with the classical emergency kit was 3.2 (scale from 1 to 6; 1 = not very confident and 6 = very confident). The following confidence scores for equipment retrieval were obtained: 5,3 for peripheral venous access, 3,0 for IO, 4,4 for intubation and 4,7 for epinephrine. Comparing nurses from the IMC and ICU, ICU nurses felt significantly more confident in preparing equipment for intubation (*p* < 0.001) and epinephrine (0.01), but this was not true for peripheral venous access, intraosseous access or overall handling with the kit.

### Time Required for Equipment Retrieval

**Figure 2:**
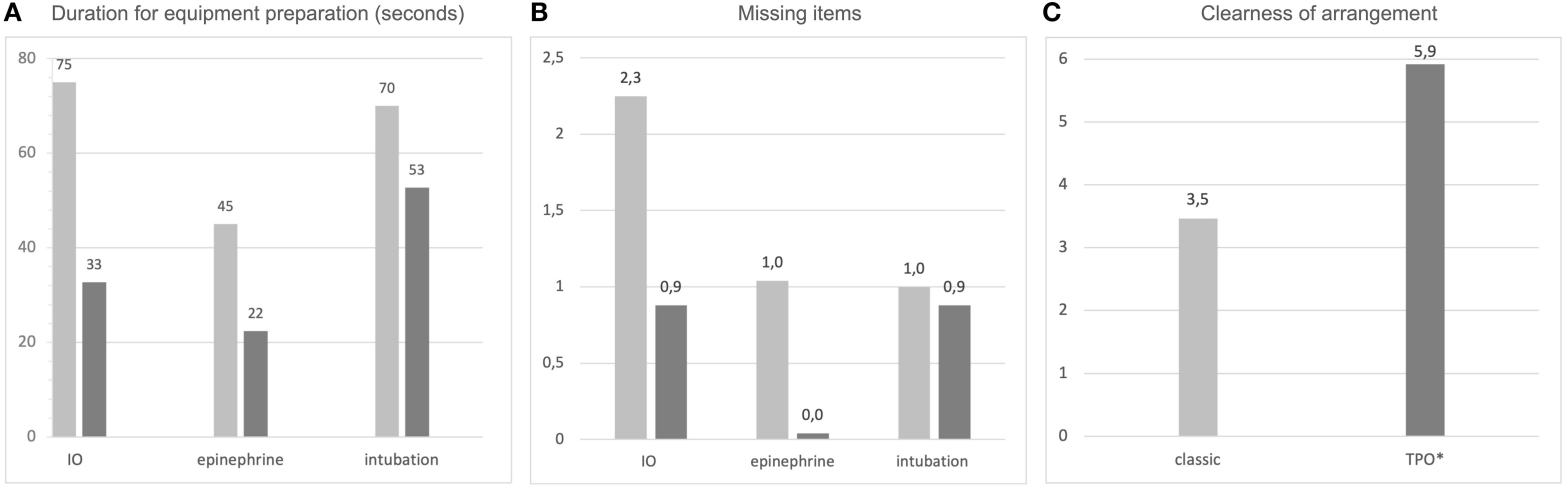
Comparison of the classical emergency kit with the task-based package-organized emergency kit according to **(A)** the time required to prepare equipment for IO access, epinephrine and intubation (numbers are given in seconds); **(B)** missing items in equipment preparation and **(C)** overall clearness of arrangement (scale from 1 to 6, with 1 = very unclear and 6 = very clear arrangement).

Compared to the classical emergency kit, times required to retrieve equipment for IO access, intubation and epinephrine were significantly shorter with the TPO kit: IO 75 vs. 33 s (*p* < 0.001), intubation 70 vs. 53 s (*p* = 0.001) and epinephrine 45 vs. 22 s (*p* < 0.001). Details are reported in Table 3; Figure 2.

**Table 3:** Statistical comparison of the classical with the task-based package-organized emergency backpack.

		**Mean**	**Standard deviation**	***p*-value**	**CI lower limit**	**CI upper limit**
Duration	IO classical	74,67	36,45	<0.001^*^	26,33	57,59
	IO TPO	32,71	19,72			
	Intubation classical	70	28,35	0.043^*^	7,47	27,11
	Intubation TPO	52,71	16,17			
	Adrenaline classical	45,04	16,84	<0.001^*^	15,37	29,88
	Adrenaline TPO	22,42	6,9			
Missing Items	IO classical	2,25	1,19	<0.001^*^	0,86	1,87
	IO TPO	0,88	0,34			
	Intubation classical	1	0,59	0,54	−0,29	0,54
	Intubation TPO	0,88	0,85			
	Adrenaline classical	1,04	0,69	<0.001^*^	0,69	1,31
	Adrenaline TPO	0,04	0,2			
Arrangement	clearness classical	3,46	1,22	<0.001^*^	−3	−1,94
	clearness TPO	5,92	0,28			

*Data regarding duration, missing items and overall clearness of arrangement of the classical emergency kit and the TPO emergency kit*.

* *Significant at p < 0.05*.

### Missing Items

The number of missing items was higher using the classical kit compared to the TPO kit (Figure 2): IO 2.3 vs. 0.9 missing items (*p* < 0.001) and epinephrine 1 vs. 0.04 (*p* < 0.001). However, the difference between the number of missing items for intubation was not significant when comparing the two kits (1 vs. 0.9—not significant).

### Clear Arrangement

Arrangement of the emergency equipment was rated as 3,5 for the classical kit and 5,9 for the TPO kit on a scale from 1 to 6 with 1 = poorly arranged and 6 = very clearly arranged (Figure 2). This result was statistically significant (*p* < 0.001).

### Frequency of Familiarization

There was no correlation between the frequency of familiarization with the classical emergency kit and the duration of equipment retrieval. This was true for the use of the classical as well as for the TPO kit and for all three types of assessments (IO access, intubation and epinephrine).

### Previous Experience

Regarding the classical kit, the more years of experience in neonatal care the employees had, the faster they were in retrieval of equipment for epinephrine (*p* = 0.04). However, no such correlation was noted with the TPO emergency kit.

Using the TPO kit, the retrieval of intubation equipment was significantly faster in employees with more experience in neonatal care (*p* = 0.001). This was not true for the classical emergency kit. Details are provided in Table 4.

**Table 4:** Correlation of previous experience and self-rating with equipment retrieval times.

**Duration**	**Years of experience**	**Years in this hospital**	**Self-evaluation overall handling**	**Self-evaluation IO**	**Self-evaluation intubation**	**Self-evaluation adrenaline**
IO—classical	0,37	0,11	0,43	0,08	0,007^*^	0,006^*^
IO—TPO	0,41	0,62	0,85	0,83	0,06	0,87
Intub—classical	0,08	0,13	0,56	0,79	0,04^*^	0,09
Intub—TPO	0,001^*^	0,001^*^	0,88	0,75	0,04^*^	<0,001^*^
adr—classical	0,04^*^	0,06	0,95	0,25	0,27	0,52
adr—TPO	0,15	0,62	0,35	0,29	0,03^*^	0,03^*^

*Correlation of previous experience and rating of self-evaluation scales of equipment preparation with the durations of different equipment preparation*.

* *Significant at level of p < 0.05*.

### Self-Evaluation

Personnel who rated their abilities in the overall handling of the emergency kit higher were not significantly faster in equipment retrieval (IO, intubation and epinephrine). Details on IO, intubation and epinephrine retrieval are presented in Table 4.

### ICU vs. IMC

The time required for IO retrieval was significantly shorter among ICU personnel compared with IMC personnel (classical kit: 55 vs. 94 s, *p* = 0.006; TPO: 24 vs. 41 s, *p* = 0.04). All other parameters (time required for intubation/epinephrine retrieval or missing items) did not differ in ICU compared to IMC staff.

## Discussion

In stressful situations, even the simplest tasks might become extremely challenging. Therefore, in emergency events it should be made easy for medical teams to find and retrieve the required emergency equipment as fast as possible (5, 6). However, this is not only relevant for mobile emergency kits (though events outside the “home ward” are supposed to be the most stressful and challenging). This is true for all emergency carts, resuscitation rooms and, in general, all sites where critical events might occur. Furthermore, an excellent way to train and evaluate the arrangement of equipment is to simulate critical events in real-life settings (*in-situ* simulation training), which should be done in regular intervals to be able to dynamically adapt to changing conditions or treatment strategies.

The consideration of human factors in emergency backpacks that might impede the retrieval of material is of paramount importance (7, 8). These factors include, among others, the appropriate choice of backpack/trolley type and storage bags (size, color, type of closure systems), clear labeling of the bags and arrangement of the bags within the backpack. A successful example on how to include human factors in equipment arrangement and to evaluate the approach using *in-situ* simulation is provided by Lefebvre et al., describing the process of re-designing their neonatal surgical crash cart (9). To our knowledge, no study has focused on how to arrange equipment in a neonatal emergency backpack and how to consider human factors to optimize equipment preparation.

In this study we used simulation to compare the classical neonatal emergency backpack with the TPO kit and showed that equipment retrieval times for three different tasks (IO, intubation and epinephrine administration) were significantly shorter, there were fewer missing items and subjective ratings of “overall clearness” were significantly higher in the TPO kit. As a single exception, the number of missing items for intubation did not differ significantly between classical and TPO kit. This, however, was to be expected given that the “airway”-packages are very similar and, in both approaches, there is no requirement for additional equipment.

It seems obvious that the requirement of several storage bags in the classical kit that have to be identified, opened and searched for certain equipment is challenging and time-consuming. The advantage of the TPO approach clearly lies within the underlying idea that everything needed for one specific task is packed together as a set, and no additional storage bags are needed. This notion is supported even more by the use of the small subsets within the task-based bags, i.e., the equipment for one attempt of peripheral venous access is packed together separately and ready to hand.

Based on the results of this study, we conclude that neonatal emergency backpacks might preferably be arranged in a TPO-based manner rather than an ABC- or material-oriented way. As an example, we present the content and packages of our neonatal TPO emergency backpack. However, medical teams in other hospitals are asked to arrange their packages according to setting-specific requirements, treatment strategies and local customs. Further studies are required to test the TPO approach as well in carts and other emergency storage systems.

Additional factors might influence retrieval time. It is assumed that personnel are faster in equipment retrieval if they regularly familiarize themselves with the emergency backpack. Interestingly, there was no significant correlation between the frequency of self-acquaintance and the equipment retrieval time. However, this might be due to the possible answers in the questionnaire: The majority of participants chose “1–2 times every 6 months” or “1–2 times every 12 months” and the differences here might be too low to obtain significant results. To our knowledge, there is no clear data on how often personnel should familiarize themselves with emergency kits. However, in basic life support, a significant loss of effectiveness of skills, such as ventilation and chest compression, occurs as soon as four weeks after a training session (10). Hence, despite the results of this study, we still recommend regular, short-interval familiarization and training with all emergency equipment.

Within this study, when using the classical emergency backpack, personnel with more neonatal working experience showed faster retrieval times for epinephrine equipment. Interestingly, this was not the case when the TPO kit was used. One might conclude that more experience is required to reach fast retrieval times for the classical kit, and that this is not necessary for the TPO kit due to the easier storage approach. However, the opposite findings were noted for the retrieval time for intubation. Specifically, more experienced personnel retrieved equipment for intubation faster when using the TPO kit, but this finding was not significant for the classical kit. Based on the results of this study, we conclude that years of experience are not sufficient to explain the speed of equipment retrieval. One explanation might be that regular, short-interval training is more important than years of experience in the case of emergency kit handling.

The impact of the subjective self-evaluation in the handling of the emergency kit was analyzed to test the assumption that personnel with higher self-ratings might also show faster retrieval times. Surprisingly, personnel with higher ratings in the self-evaluation of “overall handling” of the emergency kit were not faster than personnel with lower ratings. This finding reflects that an individual's perception about their own preparedness for an emergency event might be incorrect. Consequently, in addition to optimizing equipment organization with TPO packages, we recommend obligatory training for the personnel regardless of self-perceptions of familiarity or experience.

In the case of an emergency event that occurs outside the NICU, standardized procedures are in order in our institution so that ICU personnel running to the site of the event also bring along the emergency backpack. Therefore, one might assume that ICU personnel would be more familiar with working with the emergency backpack and thus might show faster retrieval times. Interestingly, this was not the case in our study. When comparing ICU to IMC personnel, nearly all retrieval times as well as the number of missing items were not significantly different. One possible explanation for this finding might be that although emergency events occur more frequently in ICUs, they are still rare events. Therefore, ICU personnel might wrongly assume that they are “trained” sufficiently by real-life events. Additionally, given that ICU personnel had more years of neonatal experience, incorrect self-evaluation of preparedness for emergency events might further aggravate this issue. Consequently, we recommend that all personnel, including (or maybe especially) ICU personnel, regularly undergo training for the handling of emergency kits and equipment retrieval.

## Limitations

This study was conducted using *in-situ* simulation, but without the use of a resuscitation scenario (the participants were merely asked to retrieve the according equipment). Also, it was performed exclusively in one single institution. Further, only a subset of tasks of the TPO-bags were tested and which kit was used first was not randomized.

The results might differ in a resuscitation-based simulation scenario, a non-simulation (real life) setting or in other settings/countries.

## Conclusion

The TPO approach in the neonatal emergency backpack seems to lead to faster retrieval times of emergency equipment, fewer missing items and clearer arrangement compared to the classical ABC or material-oriented storage approach.

## Data Availability Statement

The raw data supporting the conclusions of this article will be made available by the authors, without undue reservation.

## Ethics Statement

The studies involving human participants were reviewed and approved by Ethics Committee of the Medical University Vienna, Austria. The patients/participants provided their written informed consent to participate in this study.

## Author Contributions

LS: conceptualization, methodology, investigation, formal analysis, data curation, writing—review & editing, and project administration. MH-D: conceptualization, methodology, investigation, formal analysis, and writing—review & editing. KK-S and AB: conceptualization, methodology, formal analysis, and writing—review & editing. ES: conceptualization, methodology, formal analysis, data curation, writing—original draft, visualization, and project administration. All authors contributed to the article and approved the submitted version.

## Conflict of Interest

ES is a neonatal consultant at the Medical University Vienna and managing partner/CEO of SIMCharacters Training GmbH (Austria), a company providing medical simulation training in German-speaking countries. However, there is no financial or other related benefit in relation to this manuscript. The remaining authors declare that the research was conducted in the absence of any commercial or financial relationships that could be construed as a potential conflict of interest.

## Publisher's Note

All claims expressed in this article are solely those of the authors and do not necessarily represent those of their affiliated organizations, or those of the publisher, the editors and the reviewers. Any product that may be evaluated in this article, or claim that may be made by its manufacturer, is not guaranteed or endorsed by the publisher.
